# Gadoxetic Acid in MRI: A Five-Year Experience at a High-Complexity Hospital in Colombia

**DOI:** 10.7759/cureus.58150

**Published:** 2024-04-12

**Authors:** Jonathan Pimiento Figueroa, Johan Sebastian Lopera Valle, Ana M Gomez Urrego, Vanessa García Gómez, Mateo Gonzalez, Claudia Huertas Duran

**Affiliations:** 1 Department of Radiology, Servicios de Salud San Vicente Fundación, Medellín, COL; 2 Department of Radiology, Clinica Las Americas, Medellín, COL; 3 Department of Radiology, Hospital Pablo Tobón Uribe, Medellín, COL

**Keywords:** bile ducts, liver cell, adenoma, focal nodular hyperplasia, contrast media, liver diseases, magnetic resonance imaging

## Abstract

Objective

The objective of the study was to evaluate the use of the hepatospecific contrast agent, gadoxetic acid, for MRI in patients at a high-complexity hospital in Medellin, Colombia, from 2016 to 2022.

Materials and methods

This was an observational, descriptive, and retrospective cross-sectional study involving patients who had undergone MRI with gadoxetic acid from February 2016 to January 2022. The MRI studies were interpreted by two radiologists specializing in body imaging, each with at least 10 years of experience. The medical records of the identified patients were reviewed. Quantitative variables were presented using either means and standard deviations or medians and interquartile ranges, depending on the distribution of the variables. Qualitative variables were represented through absolute and relative frequencies.

Results

A total of 100 pharmacy records were collected, leading to a final sample of 75 patients aged between three and 91 years. The primary reason for imaging was to assess focal liver lesions in 58 patients (77.3%), with bile duct injury being the second most common indication in 16 patients (21.3%). A diagnostic alteration was noted in 69.3% of cases (52 patients). Among the 58 focal liver lesions analyzed using a hepatospecific agent, 31 cases (53.4%) were diagnosed as focal nodular hyperplasia.

Conclusion

Our study reinforces the clinical value of gadoxetic acid-enhanced MRI in refining diagnostic assessments, particularly in cases involving bile duct and focal hepatic lesions.

## Introduction

Intracellular/hepatobiliary gadolinium-based contrast agents differ from extracellular ones due to their unique properties. They diffuse into the extracellular space like extracellular contrasts, then use membrane proteins to enter hepatocytes and are excreted through bile ducts, similar to bilirubin. This contrast entering hepatocytes enhances the appearance of parenchyma significantly, improving the detection, characterization, and functional assessment of focal lesions in the liver. Moreover, their excretion through the biliary pathway allows for more accurate anatomical characterization [1].

Considering all the points mentioned above, these agents are seen as a valuable resource for tackling diagnostic issues in patients with focal hepatic lesions showing atypical features or those considered uncertain by other diagnostic techniques. They aid in evaluating lesions that have bile ducts, such as dysplastic nodules in cirrhotic patients and focal nodular hyperplasia (FNH) in non-cirrhotic patients [2].

There is evidence supporting the effectiveness of hepatobiliary contrast agents in evaluating hepatic lesions that are inconclusive with other diagnostic methods and in characterizing bile duct lesions. However, the utilization rate of these agents in Colombia remains low, even in highly advanced hepatobiliary referral centers. Therefore, this study aims to describe the experience of using the hepatospecific contrast agent, gadoxetic acid, for magnetic resonance imaging (MRI) in patients treated at a high-complexity hospital in Medellin, Colombia.

## Materials and methods

This was an observational, descriptive, and retrospective cross-sectional study. A search was performed in the hospital's pharmaceutical registry to find patients who were administered gadoxetic acid (Gd-EOB-DTPA, Primovist®) from February 2016 to January 2022. Confirmation of MRI acquisition was done through the institutional picture archiving and communication system (PACS) (Enterprise Imaging Platform; Agfa-Gevaert N.V., Mortsel, Belgium). Additionally, the medical records of these patients were reviewed to collect relevant variables. The study obtained approval from the Ethics Committee of Pablo Tobón Uribe Hospital, Medellín, Colombia (approval number: 062022), and was conducted in adherence to ethical principles for research, following the Declaration of Helsinki and Resolution 008430 of 1993 from the Ministry of Health of Colombia.

A convenience non-probabilistic sampling was conducted of all patients meeting the single inclusion criterion: those who had an MRI study at Hospital Pablo Tobón Uribe between February 2016 and January 2022, using gadoxetic acid. All MRI studies were done on Siemens 1.5 and 3 Tesla MRI scanners (Siemens Healthineers AG, Erlangen, Germany), with gadoxetic acid Gd-EOB-DTPA (Primovist) administered at a dose of 0.1 ml/kg from a preloaded syringe with a concentration of 0.25 mmol/ml. Interpretation of MRI studies was done by two radiologists specializing in body imaging, each with at least 10 years of experience.

Quantitative variables are typically presented using means ± SD. Qualitative variables are described using absolute and relative frequencies.

## Results

During the study period, we collected 100 pharmacy records. Upon examination, we identified 25 patients with multiple occurrences, resulting in a final cohort of 75 patients for evaluation. Among them, 53 were female (70.6%), ranging in age from three to 91 years with an average age of 41 years (SD: 17). Only six patients had cirrhosis (8%), and one had undergone a liver transplant. The primary objective of the MRI was to categorize focal liver lesions previously deemed inconclusive by another diagnostic technique, constituting 77.3% (58 patients) of the cases (Table 1). Of the 58 focal liver lesions examined with a hepatospecific agent, 31 cases (53.4%) were identified as focal nodular hyperplasia. In 11 (68.7%) out of 16 patients suspected of bile duct injury, confirmation of fistula, bilioma, and/or stenosis was achieved during the assessment of the bile duct (Table 1).

**Table 1:** Clinical characteristics of patients undergoing MRI with gadoxetic acid. Data presented as n (%) except for age, which is presented as mean ± SD

Characteristices	N=75
Female gender, n (%)	53 (70.6%)
Age (years), mean ± SD	41 ± 17
MRI indication, n (%)	
Focal liver lesion	58 (77.3%)
Bile duct injury	16 (21.3%)
Other	1 (1.3%)
Diagnosis change	52 (69.3%)
MRI focal lesion finding, n (%)	N=58
Benign	48 (82.7%)
Malignant	5 (8.6%)
Indeterminate	5 (8.6%)
Benign focal lesion, n (%)	N = 48
Focal nodular hyperplasia	31 (64.5%)
Hepatocellular adenoma	10 (20.8%)
Hemangioma	4 (8.3%)
Hepatic pseudo lesion	3 (6.2%)
Malign focal lesion, n (%)	N = 5
Hepatocellular carcinoma	3 (60%)
Metastasis	2 (40%)
MRI cholangiography, n (%)	N=16
Bile duct injury	11 (68.7%)
Non-bile duct injury	4 (25%)
Indeterminate	1 (6.2%)

The use of MRI with a liver-specific contrast agent led to changes in the original MRI diagnosis (using extracellular contrast agents) in 52 patients (69.3%), with bile duct abnormalities detected in 14 patients and localized liver lesions identified in 38 patients. This indicates an alteration in the initial MRI diagnosis for 87.5% of patients with suspected bile duct injury and 65.5% of patients with indications of localized liver lesions on MRI (Table 2).

**Table 2:** Distribution of indication type according to the change in diagnosis.

Indication	Diagnosis change		Total, n
	No, n (%)	Yes, n (%)	
Focal liver lesion	20 (34.5%)	38 (65.5%)	58
Bile duct injury	2 (12.5%)	14 (87.5)	16
Other	1 (100%)	0	1

## Discussion

Gadoxetic acid, a contrast agent for MRI, plays a crucial role in evaluating hepatobiliary diseases. It moves through vascular and extravascular spaces in the dynamic phase, then gets taken up by hepatocytes and excreted into bile ducts in the hepatobiliary phase. Diagnostic studies using gadoxetic acid are non-invasive and provide significant advantages in evaluating liver and biliary system disorders [2,3]. Gadoxetic acid exhibits a safety profile akin to American College of Radiology group II gadolinium-based contrast agents concerning hypersensitivity reactions and nephrogenic systemic fibrosis [3,4].

This imaging technique allows for precise characterization of focal liver lesions, such as hepatocellular carcinoma and metastases, as well as accurate evaluation of bile duct abnormalities like strictures or fistulas. By aiding in early detection, precise diagnosis, and treatment planning, non-invasive examinations with gadoxetic acid help enhance patient outcomes, decrease procedural risks, and optimize healthcare resource utilization. Additionally, the safety profile and tolerability of gadoxetic acid-enhanced MRI make it a preferred option for both patients and clinicians, highlighting its crucial role in contemporary hepatobiliary diagnostics [3-5].

The use of the gadoxetic acid contrast agent in a high-complexity hospital, particularly 100 times over a five-year period in our study, prompts a comparison with its usage in other hospitals worldwide. For instance, Jang et al. (2022) conducted a comprehensive study with 2230 patients who underwent gadoxetic acid-enhanced MRI [6]. Their research aimed to identify risk factors linked to transient severe motion artifacts during the arterial phase of the imaging procedure. The substantial cohort size in the study emphasizes the extensive use of gadoxetic acid-enhanced MRI in clinical settings globally, underscoring its significance as a valuable diagnostic tool for hepatobiliary imaging. This variability highlights the diverse utilization patterns of gadoxetic acid across various healthcare systems and geographic locations, influenced by factors like resource availability, clinical practices, and patient demographics [4,5].

The use of gadoxetic acid in MRI has been found to be valuable in clinical practice. Our study showed a significant impact on diagnostic accuracy, leading to a notable change in the working diagnosis for many patients. Specifically, MRI with a liver-specific contrast agent changed the initial MRI diagnosis (with extracellular contrast agents) for nearly seven out of 10 patients (69.3%), uncovering bile duct abnormalities in 68.7% (n=11) of patients with suspected bile duct injury. This highlights the superior capability of gadoxetic acid-enhanced MRI in detecting and characterizing biliary system abnormalities. The efficacy of gadoxetic acid in identifying biliary tract lesions has been extensively studied and proven to be highly valuable in clinical practice [2,7]. 

In patients with focal hepatic lesions, the working diagnosis was altered in 65.5% of cases (38 patients), highlighting the diagnostic value of gadoxetic acid in assessing and outlining focal hepatic pathologies. Gadoxetic acid's hepatospecific properties allow improved visualization and characterization of hepatic lesions, leading to more accurate diagnoses and enhanced patient management strategies [2,8,9]. FNH and adenomas are two common liver lesions affecting patients with similar epidemiologic profiles. The sensitivity of MRI with an extracellular contrast agent in diagnosing FNH varies widely, from 20% to 80%. In contrast, gadoxetic acid-enhanced MRI shows high accuracy in diagnosing these lesions [2,10,11,12]. Among the 58 focal liver lesions examined with a hepatospecific agent in our study, 31 cases (53.4%) were primarily diagnosed as FNH, and 10 (17.2%) as hepatocellular adenoma.

The lack of extensive data on the routine utilization of gadoxetic acid in hospitals globally is a significant deficiency in the current medical literature. Although there is considerable research on the effectiveness and advantages of gadoxetic acid-enhanced MRI in specific clinical situations like liver lesions and biliary tract irregularities [7,8], there is a dearth of standardized data on its regular application across different healthcare environments worldwide. This data gap impedes a thorough comprehension of the real-world consequences, obstacles, and results linked with the widespread adoption of gadoxetic acid in everyday clinical practice. Additional research and data-gathering endeavors are crucial to address this gap in information and offer valuable insights into the broader usage trends and clinical efficacy of gadoxetic acid in diverse healthcare settings globally.

A key strength of the current study, conducted with gadoxetic acid in a high-complexity hospital in Latin America, is the novelty of the research, as there is a significant lack of prior studies on the use of gadoxetic acid in the region. By addressing this knowledge gap, the study provides valuable insights into the utilization and effectiveness of gadoxetic acid-enhanced MRI in a Latin American healthcare context. Additionally, the high-complexity hospital setting enhances the study by offering access to advanced imaging technologies, specialized medical expertise, and a diverse patient population. This environment enables thorough investigations into the diagnostic capabilities, clinical outcomes, and patient experiences related to gadoxetic acid in hepatobiliary imaging.

This study has certain limitations, including being a single-center study with a descriptive and retrospective nature, which could restrict the depth and specificity of findings when compared to more intricate study designs. This limitation could impact establishing causality or definitive conclusions on gadoxetic acid's effectiveness in clinical practice. Another weakness is the small sample size and limited variables included in the study. Future research with larger sample sizes, analytical designs, and a broader range of variables could strengthen the evidence base and enhance understanding of gadoxetic acid's role in clinical practice.

## Conclusions

Our research highlights the clinical significance of gadoxetic acid-enhanced MRI in enhancing diagnostic assessments, particularly for bile duct and focal hepatic lesions. Additional research could elucidate the precise diagnostic advantages and clinical outcomes associated with gadoxetic acid in MRI, ultimately improving patient care and treatment decisions in Latin America.
